# Protective effect of α7 nicotinic acetylcholine receptor activation on experimental colitis and its mechanism

**DOI:** 10.1186/s10020-022-00532-2

**Published:** 2022-09-04

**Authors:** Wenyuan Pu, Zhenzi Su, Junaid Wazir, Chen Zhao, Lulu Wei, Ranran Wang, Qiyi Chen, Saifang Zheng, Shaoyi Zhang, Hongwei Wang

**Affiliations:** 1 State Key Laboratory of Analytical Chemistry for Life Science and Jiangsu Key Laboratory of Molecular Medicine, Medical School of Nanjing University, Nanjing, 210093 China; 2 The Affiliated Suqian Hospital of Xuzhou Medical University and Nanjing Drum Tower Hospital Group Suqian Hospital, Suqian, 223800 China; 3 Department of Colorectal Disease, Intestinal Microenvironment Treatment Center, Shanghai Tenth People’s Hospital, Tenth People’s Hospital of Tongji University, Shanghai, 200072 China; 4 Department of Pathology, Ruijin Hospital, Shanghai Jiaotong University School of Medicine, 197 Ruijin Second Road, Shanghai, 200025 China

**Keywords:** IBD, α7nAChR, NF-κB, MAPK, Cholinergic anti-inflammatory pathway

## Abstract

**Background:**

Inflammatory bowel disease (IBD) is a common chronic remitting disease with no satisfactory treatment. The aim of this study was to investigate the protective effect of α7 nicotinic acetylcholine receptor (α7nAChR), and to determine the underlying mechanism of its activity.

**Methods:**

The expression and distribution of α7nAChR in the intestinal tissue of patients with ulcerative colitis and Crohn’s disease were analyzed. The effects of vagal excitation on murine experimental colitis were investigated. The colitis model was induced in C57BL/6 mice by the administration of 3% dextran sulfate sodium (DSS). The therapeutic group received treatment with the α7nAChR agonist PNU-282987 by intraperitoneal injection.

**Results:**

Our results showed that there was significantly increased expression of α7nAChR in colitis and Crohn’s disease intestinal tissue, and its expression was mainly located in macrophages and neutrophils, which were extensively infiltrated in the disease status. Treatment with an α7nAChR agonist potently ameliorated the DSS-induced illness state, including weight loss, stool consistency, bleeding, colon shortening, and colon histological injury. α7nAChR agonist exerted anti-inflammatory effects in DSS colitis mice by suppressing the secretion of multiple types of proinflammatory factors, such as IL6, TNFα, and IL1β, and it also inhibited the colonic infiltration of inflammatory cells by blocking the DSS-induced overactivation of the NF-κB and MAPK signaling pathways. Mechanistically, activation of α7nAChR decreased the number of infiltrated M1 macrophages in the colitis intestine and inhibited the phagocytosis ability of macrophages, which were activated in response to LPS stimulation.

**Conclusion:**

Thus, an α7nAChR agonist ameliorated colonic pathology and inflammation in DSS-induced colitis mice by blocking the activation of inflammatory M1 macrophages.

## Introduction

Inflammatory bowel disease (IBD) is a group of chronic inflammatory disorders of the colon and small intestine. The incidence of IBD is increasing gradually in Western countries with a high need for novel therapeutic interventions. There are two major types of IBD, ulcerative colitis (UC), which is limited to the colonic mucosa, and Crohn’s disease (CD), which can affect any segment of the gastrointestinal tract (Sales-Campos 2017). The pathological features of IBD include chronic inflammation of the gastrointestinal (GI) tract; prolonged inflammation results in damage to the GI tract, which is manifested by mucosal injuries, body weight loss, altered stool consistency, blood feces, and colonic shortening (Basso 2014). Although the pathological mechanisms of IBD are still not fully understood, it is widely believed that the pathogenesis of this disease is the result of the dysfunction of mucosal immune responses, caused by a combination of environmental factors, genetic specificity, and the effect of intestinal flora (Ng 2013).

Recent studies have indicated that the neural and immune systems interact with each other to control immune hemostasis (Colling et al. 2021). The neuroendocrine system signals to the immune system via the release of hormones and neurotransmitters that regulate cellular activity via these receptors. Immune cells express various types of receptors for hormones or neurotransmitters. Acetylcholine (ACh), the first neurotransmitter that has been discovered, is a well-known multifunctional molecule in many sites of either neuronal or nonneuronal tissues. Recent reports have suggested that the acetylcholine nerve can modulate the immune response and control inflammation through a cholinergic anti-inflammatory pathway (CAP) (Wedn et al. 2021; Alen 2022). In this process, nicotinic acetylcholine receptors of the α7 subtype (α7nAChRs) play a key role in controlling immune function and proinflammatory responses. α7nAChRs are cationic channels of the Cys-loop receptor family. As one of the most abundant nAChRs in the central nervous system, α7nAChRs contribute to cognitive functioning, sensory information processing, attention, working memory, and reward pathways (Zanetti 2016). Apart from neuron cells, α7nAChR has been found to be widely expressed in various types of immune cells, including macrophages, dendritic cells (DCs), lymphocytes, neutrophils, and enterocytes, endothelial cells, and microglia. Activation of the CAP could block the rogue inflammatory response and treat a number of diseases, including life-threatening sepsis, rheumatoid arthritis, inflammation-mediated renal injury, and respiratory inflammatory diseases such as asthma and chronic obstructive pulmonary disease. In some studies, the use of a subset of cholinergic receptor antagonistic peptides and α7nAChR knockout mice enhanced the secretion of inflammatory factors by macrophages and aggravated intestinal inflammation in mice. Interestingly, the anti-inflammatory effect of vagus nerve stimulation in the intestinal tract has nothing to do with the spleen and T cells (Wang et al. 2002; Matteoli et al. 2014). However, the functional role of the CAP in intestinal inflammation, especially in IBD, still needs to be defined.

Intestinal macrophages are key components for the maintenance of tissue integrity because they can distinguish innocuous antigens and potential pathogens to maintain gut immune homeostasis, and the activation of Toll-like receptors (TLRs) plays a critical role in this process. Aberrant activation of TLR4 by gut bacteria-released LPS results in the secretion of multiple types of proinflammatory molecules, including TNF-α and IL-6, and has been found to be associated with the pathogenesis development of IBD (Fonseca-Camarillo 2009; Qi 2019). Blocking TLR4-associated nuclear factor κB (NF-κB) and mitogen-activated protein kinase (MAPK) pathway activation or recovering the polarization of macrophages from an M1-type proinflammatory phenotype to an M1-type anti-inflammatory phenotype could both alleviate experimental colitis (Tang 2019). Previous studies have well established that there is a high level of α7nAChR expression on antigen-presenting cells, such as macrophages and dendritic cells. Therefore, it is interesting to know whether regulation of CAP activation in macrophages could show any therapeutic effect in IBD.

Based on previous reports, intestinal inflammation triggers a vagal-mediated circuit, thus activating vagal motor neurons connected to the inflamed intestine. This finding provides direct neuroanatomical evidence demonstrating the existence of the vagal-mediated CAP reflex in the intestine (Cailotto 2012). However, the effects of CAP activation on IBD treatment are still under debate. Some reports have indicated that CAP has promising therapeutic potential for the treatment of IBD (Bonaz 2017), while other reports show that CAP should not be the major target for IBD anti-inflammatory therapy (Shifrin 2017). To address this question, in our study, we aimed to evaluate the therapeutic effects of CAP activation on intestinal inflammation using a DSS-induced experimental colitis mouse model. Additionally, the molecular mechanisms involved in this process will be delineated.

## Materials and methods

### Human clinical samples

Subjects were recruited from among patients referred for colonoscopy at Tongji Hospital, Shanghai, China. Biopsy specimens from the sigmoid colon of 3 patients with UC and 3 patients with CD were assessed. Biopsy samples were also obtained from 3 patients with normal mucosa (control group). Patients were diagnosed with UC and CD according to the consensus opinion on the diagnosis and treatment of IBD in China. Endoscopy and imaging were consistent with the characteristics of UC and CD and confirmed by pathology biopsy. The surgical specimens (including the intestinal segment and the lymph nodes near the lesion) were pathologically proven to be UC and CD. The age ranged from 18 to 60 years, and the clinical data were complete. The colon biopsy specimens of the normal control group were obtained from the intestines of patients with sigmoid volvulus after surgical resection. Written informed consent was obtained from all study subjects, and approval for this study was granted by the Human Research Ethics Committee of Tongji University.

### Experimental animals

Male C57BL/6 mice, aged 6–8 weeks and weighing 22–25 g, were obtained from the Model Animal Research Center of Nanjing University (Nanjing, China). These mice were housed under normal laboratory conditions and maintained at a controlled temperature of 25 °C in a specific pathogen-free standard with circadian light/dark cycles and free access to mouse food and water. All studies were conducted according to the Institutional Animal Care and Use Committee at Nanjing University.

### Induction and treatment of colitis

**Fig. 1:**
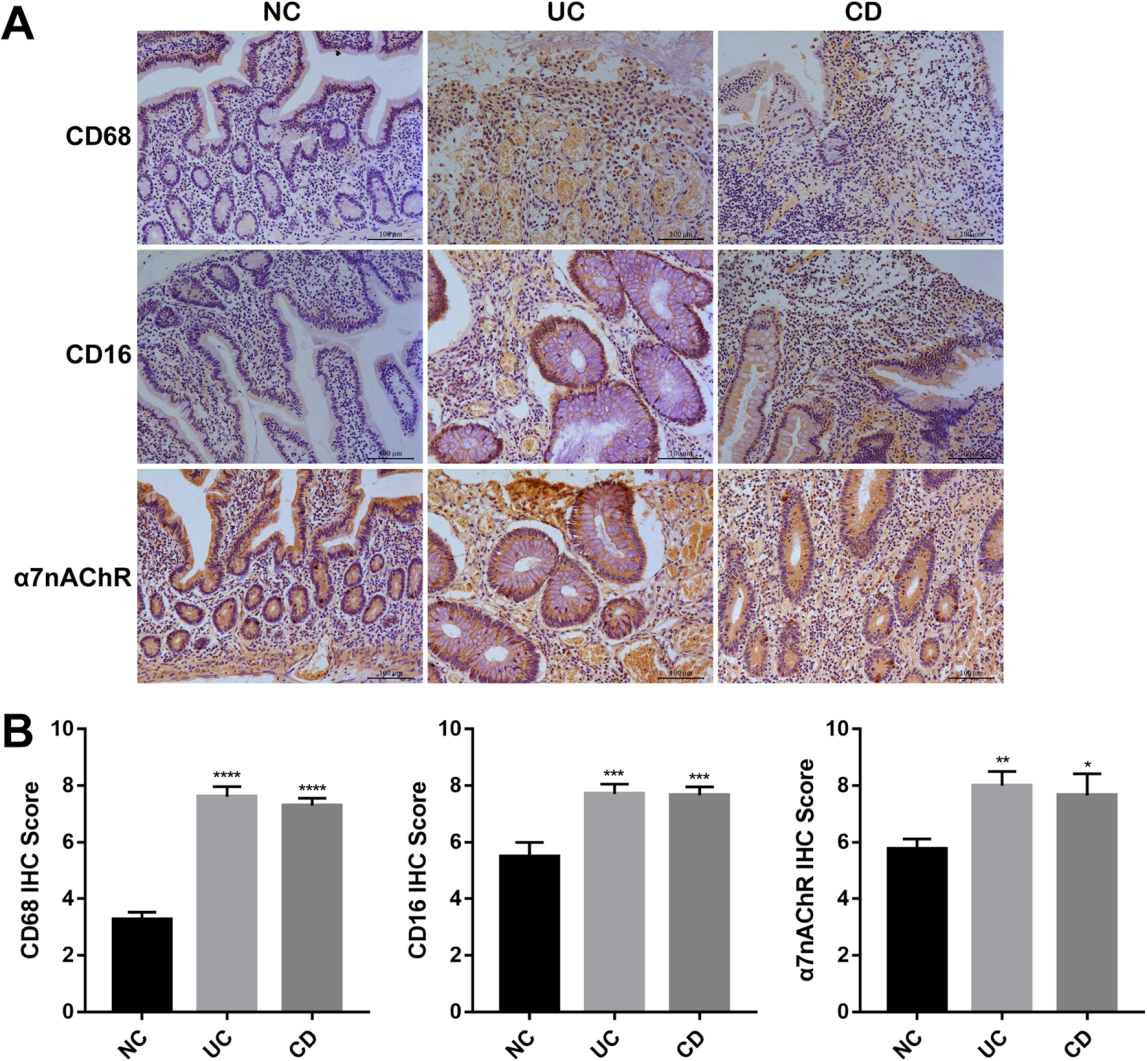
Increased α7nAChR expression and enhanced immune cell infiltration in human UC and CD colon tissues. Immunohistochemistry (IHC) of UC and CD compared with the control groups. **A** Representative images and IHC scores of the colonic tissues from normal control (NC), UC and CD patients. The expression levels of CD68, CD16 and α7nAChR were examined by IHC. Scale bar: 100 μm; **B** IHC protein expression score; *P < 0.05, **P < 0.01, ***P < 0.001, ****P < 0.0001

To evaluate the protective effects of α7nAChR agonists on colitis, an experimental chronic colitis mouse model was established by giving mice free water containing 3.0% (w/v) DSS (dextran sulfate sodium salt, molecular weight of 36 ~ 50 kDa, MP Biomedicals, LLC, SA) following a previously described protocol (Sales-Campos 2017). PNU-282987 is a selective α7nAChR agonist. PNU-282987 was diluted in saline and administered intraperitoneally, daily, at a dose of 10 mg/kg a day, according to previous dose–response experiments performed by the group. The in vivo experiments were divided into four groups, with 6 mice in each group. The healthy control group was given sterilized water for 9 days, and the mock control group received 1 mg/ml PNU-282987 (sterile water + PNU). The colitis group was given water containing 3% DSS for 9 days. The treatment group received 3% DSS-containing water for 9 days, and the intraperitoneal injection PNU-282987 (#HY-12560A, MCE) began on day 4 (Fig. 1). After 9 days, all groups of mice were sacrificed, and colon tissues were collected for further study.

### Disease clinical score, colon length, histopathological, Masson and PAS analysis

Using the disease clinical score described criteria, DSS-induced mouse colitis was scored as the disease activity index (DAI) (Wirtz 2007). The DAI evaluation criteria for the severity of body weight loss, stool consistency alteration, and bleeding were scored. Weight losses of percentages of 0, 1–5, 5–10, and 10–20 were scored as 0, 1, 2, 3, and 4, respectively. For stool consistency, 0 was scored as normal, 2 for loose stools, and 4 for diarrhea. Bleeding was scored as 0 for no blood, 2 for hemoccult and 4 for gross bleeding. Then, these scores were added, and the sum was divided by three to calculate the 0–4 DAI scores. The mice postmortem from the cecum to the anus took the entire colon and measured colon length (Zhang 2016). Colon samples were fixed in 4% paraformaldehyde and then subjected to standard histological procedures and fixed embedding. Fixed tissues were microtomized to obtain 5 μm thick sections that were later stained with hematoxylin and eosin (H&E) for microscopic analysis. In addition, Masson’s trichrome staining was carried out to measure tissue fibrosis. Goblet cells were detected by periodic acid-Schiff staining (PAS).

### Immunohistochemistry

The mice were sacrificed after being anesthetized, and the colon tissue was washed in PBS. Colon tissues were fixed with 4% paraformaldehyde and embedded in paraffin. Serial longitudinal sections were cut at a thickness of 5 μm, then deparaffinized and rehydrated. Nonspecific immunoreactivity was blocked in the slices by incubation with normal BSA serum for 1 h at room temperature, and the sections were incubated with F4/80 (#GB113373, Servicebio, Wuhan, China), Ly6G (#GB11229, Servicebio, Wuhan, China), CD68 (#GB113150, Servicebio, Wuhan, China), CD16 (#GB14026, Servicebio, Wuhan, China), α7nAChR (ab216485, Abcam, Cambridge, UK), p-P65 (#3033, Cell Signaling Technology, MA, USA) and p-STAT3 (Tyr705, #9145, Cell Signaling Technology, MA, USA) antibodies (1:100) overnight at 4 °C, followed by treatment with a streptavidin-peroxidase kit according to the manufacturer’s instructions. Sections were counterstained with H&E. As a control, a subset of sections was incubated with PBS instead of the primary antibodies (Wang 2015). The expression of protein, represented by the staining intensity at × 400 magnification, was assessed by two independent investigators who were blinded to the experimental groups.

### Cell culture and treatment

Murine RAW264.7 monocytes were cultured with Dulbecco’s modified Eagle’s medium (DMEM) (Gibco, Grand Island, NY, USA) supplemented with 10% (v/v) fetal bovine serum (Gibco) at 37 °C in a humidified incubator with 5% CO_2_. Cells were preincubated with PNU282987 (10, 50, or 100 μmol/L) for 10 min before being challenged with lipopolysaccharides (LPS) (100 ng/mL, LPS, Sigma, Louis, MO, USA) for 12 h. Cell experiments were divided into three groups, of which the control group had no LPS or PNU, the LPS group was treated with LPS, and the last group was treated with 10/50/100 PNU.

### RNA extraction and real-time PCR

The total RNA of mouse colon tissues was extracted using the Vazyme Biotech (Nanjing, China) reagent as described by the manufacturer. One microgram of total RNA was subsequently reverse-transcribed using a kit (Takara, Beijing, China) according to the manufacturer’s instructions. The housekeeping gene β-actin was used as an internal control. The mouse mRNA primer sequences for the target genes are listed in Table 1. Real-time PCR for gene expression was performed from cDNA using Power SYBR Green PCR Master Mix with an ABI Viia 7 detector system under the standard protocol in a volume of 20 μl. The relative mRNA expression of each studied gene was calculated with the comparative ΔCt method using the formula 2^−ΔΔCt^.

**Table 1:** Primers for real-time polymerase chain reaction (PCR) analysis

Genes	Forward Primer (5′–3′)	Reverse Primer (5′–3′)
β-actin	CTAAGGCCAACCGTGAAAAG	ACCAGAGGCATACAGGGACA
IL-6	ACAAAGCCAGAGTCCTTCAGAG	GGTCCTTAGCCACTCCTTCTG
TNF-α	TCCCAGGTTCTCTTCAAGGGA	GGTGAGGAGCACGTAGTCGG
IL-1β	TCCAGGATGAGGACATGAGCA	GAACGTCACACACCAGCAGGT
Ccl2	AAAAACCTGGATCGGAACCAA	CGGGTCAACTTCACATTCAAAG
Cd11c	CTGGATAGCCTTTCTTCTGCTG	GCACACTGTGTCCGAACTCA
IL-10	GGTTGCCAAGCCTTATCGGA	ACCTGCTCCACTGCCTTGCT
Retnla	CCAATCCAGCTAACTATCCCTCC	CCAGTCAACGAGTAAGCACAG
Arg1	CTCCAAGCCAAAGTCCTTAGAG	AGGAGCTGTCATTAGGGACATC
Mrc1	CTCTGTTCAGCTATTGGACGC	CGGAATTTCTGGGATTCAGCTTC
Ym1	CAGGTCTGGCAATTCTTCTGAA	GTCTTGCTCATGTGTGTAAGTGA

### Western blot

Colon samples were homogenized in RIPA lysis buffer with 1% phosphatase and a protease inhibitor cocktail (Sigma–Aldrich, St. Louis, MO, USA). The mixture homogenates were centrifuged (12,000×g, 15 min, 4 °C), and the supernatants were collected. Protein concentration was measured following the colorimetric method. Furthermore, the supernatants were mixed with 5 × SDS/PAGE sample buffer. Equal amounts of proteins (20 μg) were separated by a 10% SDS/PAGE gel and then transferred to PVDF membranes (Merck Millipore). Then, the membranes were blocked in 5% BSA buffer for 1.5 h and incubated at 4 °C overnight with specific primary antibodies against IKBα (#4812, Cell Signaling Technology, MA, USA), p-IKBα (#2859, Cell Signaling Technology, MA, USA), p-P65 (#3033, Cell Signaling Technology, MA, USA), p-ERK (Thr202/Tyr204, #4370, Cell Signaling Technology, MA, USA), p-JNK (Thr183/Tyr185, #9255, Cell Signaling Technology, MA, USA), p-P38 (#4511, Cell Signaling Technology, MA, USA) and GAPDH (BS60630, Bioworld Technology Inc, MN, USA) at a dilution of 1:000 (Cell Signaling Technology, MA, USA). After washing with TBST three times, the membranes were incubated with a secondary antibody (Cell Signaling Technology, USA). For visualization of the bands, all membranes were incubated with the immobilon western chemiluminescent HRP substrate (Millipore, USA) for the desired durations. The relative density of the signaling band for the Western blot protein was compared with the band for the housekeeping gene GAPDH in each group.

### Immunofluorescence staining

To assess the intracellular location of the NF-κB p65 subunit, RAW264.7 cells were cultured on sterile cover slips. After the specified treatment, 4% formaldehyde was diluted in PBS at room temperature for 10 min and washed in PBS three times. Then, the cells were blocked in 5% normal serum 0.25% Triton X-100 in PBS for 1 h at room temperature. Next, the cells were incubated with anti-NF-κB p65 (1:200, #8242, Cell Signaling Technology, MA, USA) overnight at 4 °C. After rinsing three times in PBS for 5 min each, the specimens were incubated in Alexa Fluor 488-labeled goat anti-rabbit secondary antibody (1:1000, Abcam, ab150077) for 30 min at room temperature in the dark and washed three times in PBS again (Huang 2019). HOECHST staining was performed to visualize the nucleus. Finally, the cover slips were mounted, and images were viewed by a Leica TCS-SP2 confocal scanning microscope (Leica, Heidelberg, Germany). The fluorescence intensity of NF-κB in the nucleus was measured by Image-Pro Plus 6.0 software in 3 fields per experimental group.

To explore M1 and M2 macrophage ratio analysis. After fixing in 4% paraformaldehyde (PFA) and embedding in paraffin, the colon tissue was dewaxed in dimethylbenzene, dehydrated in a gradient of ethanol, and then washed with deionized water. After blocking with BSA (5%) in PBS for 30 min, the sections were incubated overnight at 4 °C with CD68 and CD163 antibodies (1:100; Servicebio, Hubei, China), rinsed with PBS for 15 min, and incubated again with secondary antibodies for 30 min at ambient temperature. After incubation with the secondary antibody, DAPI solution was used to stain the nuclei under dark conditions. Finally, the stained nuclei were examined using an Olympus FV3000 confocal laser scanning microscope (Olympus, Japan, FV3000). CD68^+^ represents M1 macrophages, and CD68^+^ and CD163^+^ represent M2 macrophages.

### Assay for phagocytic activity

The phagocytic activity of RAW264.7 cells was determined using the Phagocytosis Assay Green *E. coli* (Abcam, ab235900). For confocal laser scanning microscopy, cells were cultured in six-well plates. Add 5 μL of *E. coli* slurry to all the wells. Immediately transfer the plate back to the incubator for 3 h at 37 °C, cells were washed three times with ice-cold PBS to stop phagocytosis and fixed with freshly prepared 4% PFA for 10 min. Nuclei were stained with DAPI (Beyotime, Shanghai, China) for 5 min at room temperature. An Olympus FV3000 confocal laser scanning microscope (Olympus, Japan, FV3000) was used to image the cells.

For flow cytometry analysis, cells were harvested after being incubated with green *E. coli* and then washed three times with ice-cold PBS to stop phagocytosis (Zhao et al. 2020). Cells were resuspended in PBS at a density of 1.0 × 10^6^ cells per milliliter for flow cytometry (BD FACSCalibur) analyses.

### Statistical analysis

All data are expressed as the mean ± SD from at least three independent experiments. When the distribution was normal and there was homogeneous variance, we used the parametric one-tailed unpaired Student’s t test, and ANOVA was used to compare three or more groups. P values of 0.05 or less were considered statistically significant. All analyses were performed with GraphPad Prism 7.0 software (San Diego, CA, U.S.A., version 6.07).

## Results

### Increased expression of α7nAChR and enhanced inflammatory cell infiltration were observed in human UC and CD colon tissues

To assess whether the α7nAChR signaling pathway is involved in the pathogenesis and development of clinical IBD, colon tissues from normal individuals and patients with UC and CD were compared by immunohistochemical staining (IHC). As shown in Fig. 1A and B, after analyzing the frequency of infiltrated CD68 + macrophages and CD16 + neutrophils in the intestine, we noticed that in CD or UC patients, the number of CD68 + and CD16 + cells was significantly higher than that in healthy control individuals. Measuring its tissue distribution, we found that, unlike normal individuals, infiltrated immune cells were mainly located close to the gut mucosal epithelium. In CD or UC patients, the infiltrated inflammatory cells have a wider tissue distribution, which includes the mucosal layer, submucosal layer, and muscular layer. Notably, by immunopathological staining, we also observed that the expression level of α7nAChR was significantly increased in CD and UC patients compared with normal tissues (Fig. 1A and B), which indicated that α7nAChR-mediated CAP might participate in the pathological development of IBD.

### α7nAChR agonist administration in vivo improved experimental DSS colitis

**Fig. 2:**
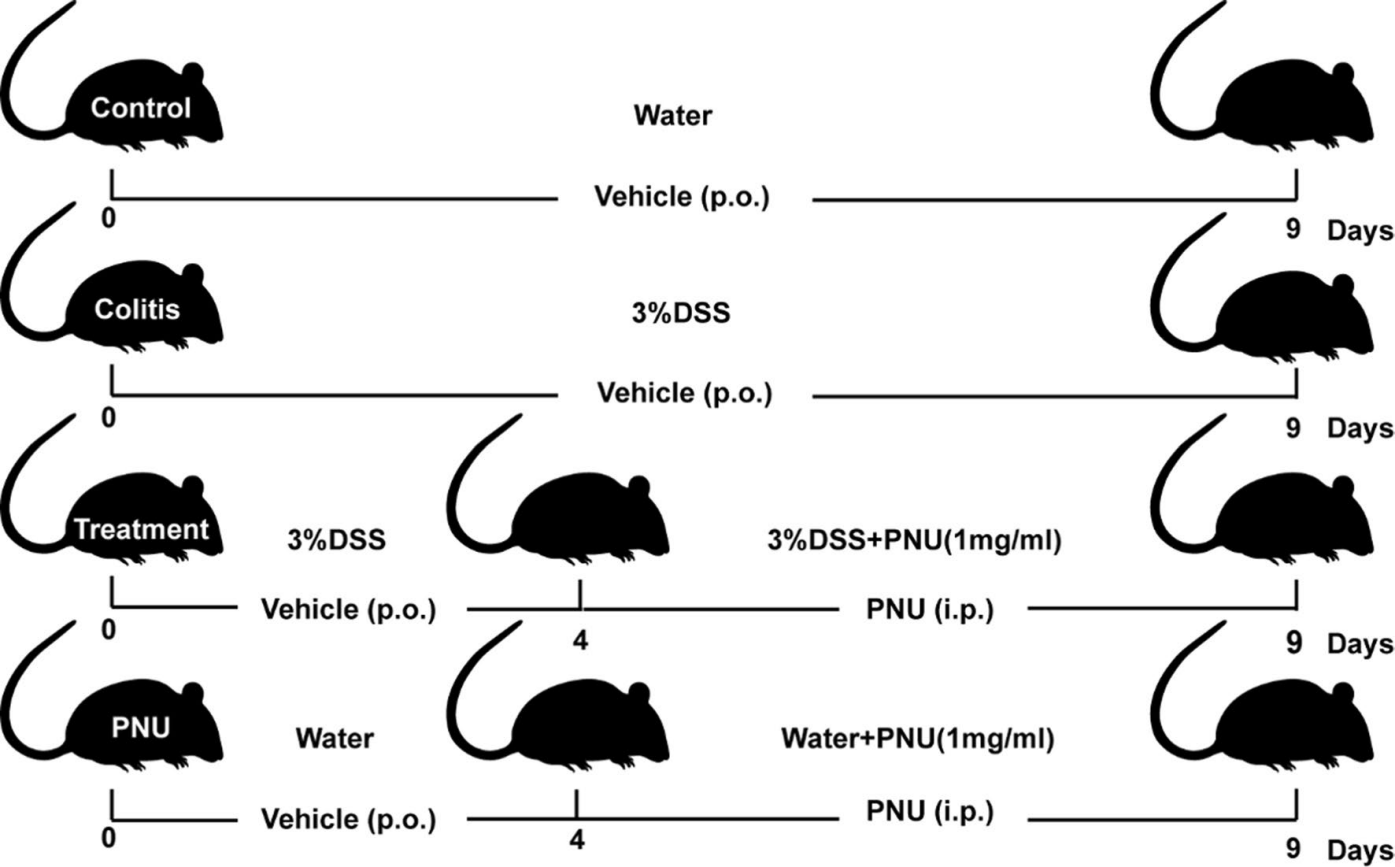
Schematic diagram of the experimental design. The mice were randomly divided into 4 groups: healthy control, colitis group, PNU treatment colitis group and PNU groups. The control group received sterile water treatment for 9 days. The colitis group was given water containing 3% DSS for 9 days. The treatment group was given 3% DSS water for 9 days, and then from the fourth day, these mice received an intraperitoneal injection of PNU-282987 (1 mg/ml) for 5 days. The PNU mock control group was given sterile water, and on the fourth day, PNU-282987 (1 mg/ml) was intraperitoneally injected

**Fig. 3:**
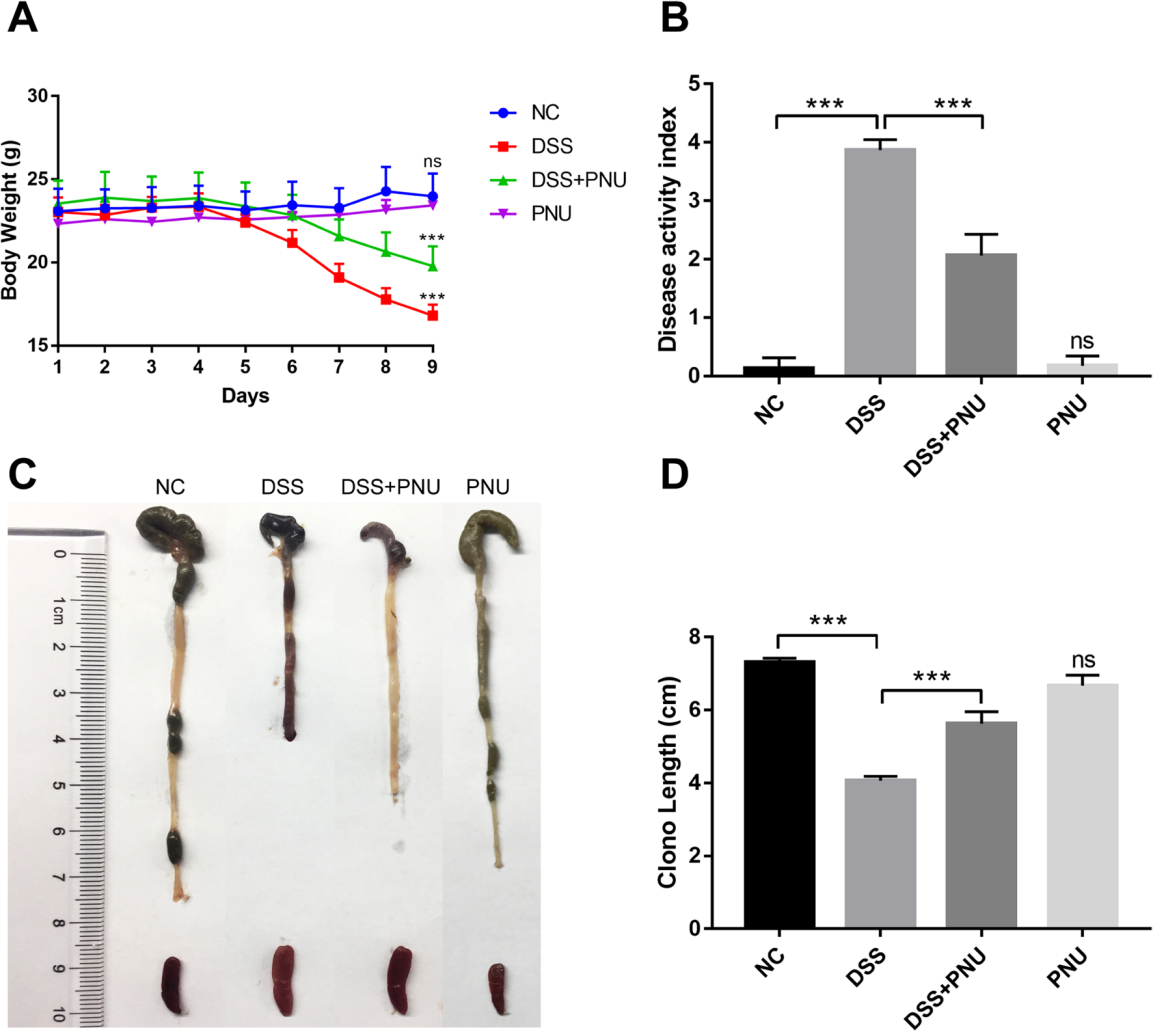
PNU treatment ameliorated DSS-induced colitis in mice. Mice received PNU-282987 treatment for 9 days. **A** Data for weight changes were recorded daily change from the starting body weight; **B** Disease activity index (DAI) was represented as average score of clinical symptoms including body weight changes, rectal bleeding and stool consistency or diarrhea; **C** and **D** Representative figure showing the colon length and spleen size for each group. The data are measured as the mean ± SD from 5 mice in each group; *P < 0.05, **P < 0.01, ***P < 0.001, compared with the IBD group

To investigate the protective effects of CAP on IBD, a DSS-induced experimental colitis mouse model was adopted, as shown in Fig. 2. Mice exposed to 3.0% DSS developed signs of colitis, and DSS administration caused severe diarrhea accompanied by extensive disease characterized by body weight loss, enhanced clinical activity score, and colon length decrease compared with the control groups, which experienced significant colitis symptoms over the observation period. In vivo treatment of colitis mice with the α7nAChR agonist PNU-282987 significantly alleviated DSS-induced colitis. The systemic pathological signs of colitis as assessed by animal weight loss and colon length were clearly attenuated (Fig. 3A), accompanied by an improvement in the severity of diarrhea and stool consistency and bleeding, combined with the clinical activity score of colitis. The overall DAI was significantly reduced compared with the experimental groups and mock treatment control groups (Fig. 3B). Colon length is an indirect and reproducible surrogate for the severity of colonic inflammation. The mice challenged with DSS exhibited a significant reduction in colon length compared to that of water control animals (Fig. 3C and D), and administration of PNU-282987 significantly reversed DSS-induced colon shortening (Fig. 3C and D). There was no significant difference in spleen weight. At the same time, using only PNU-282987 alone had no effect on the colorectal tissues of mice (Fig. 3). These results demonstrated that the activation of CAP with PNU in vivo could significantly alleviate the symptoms of experimental colitis.

### α7nAChR activation alleviated colon histopathological changes in experimental colitis

**Fig. 4:**
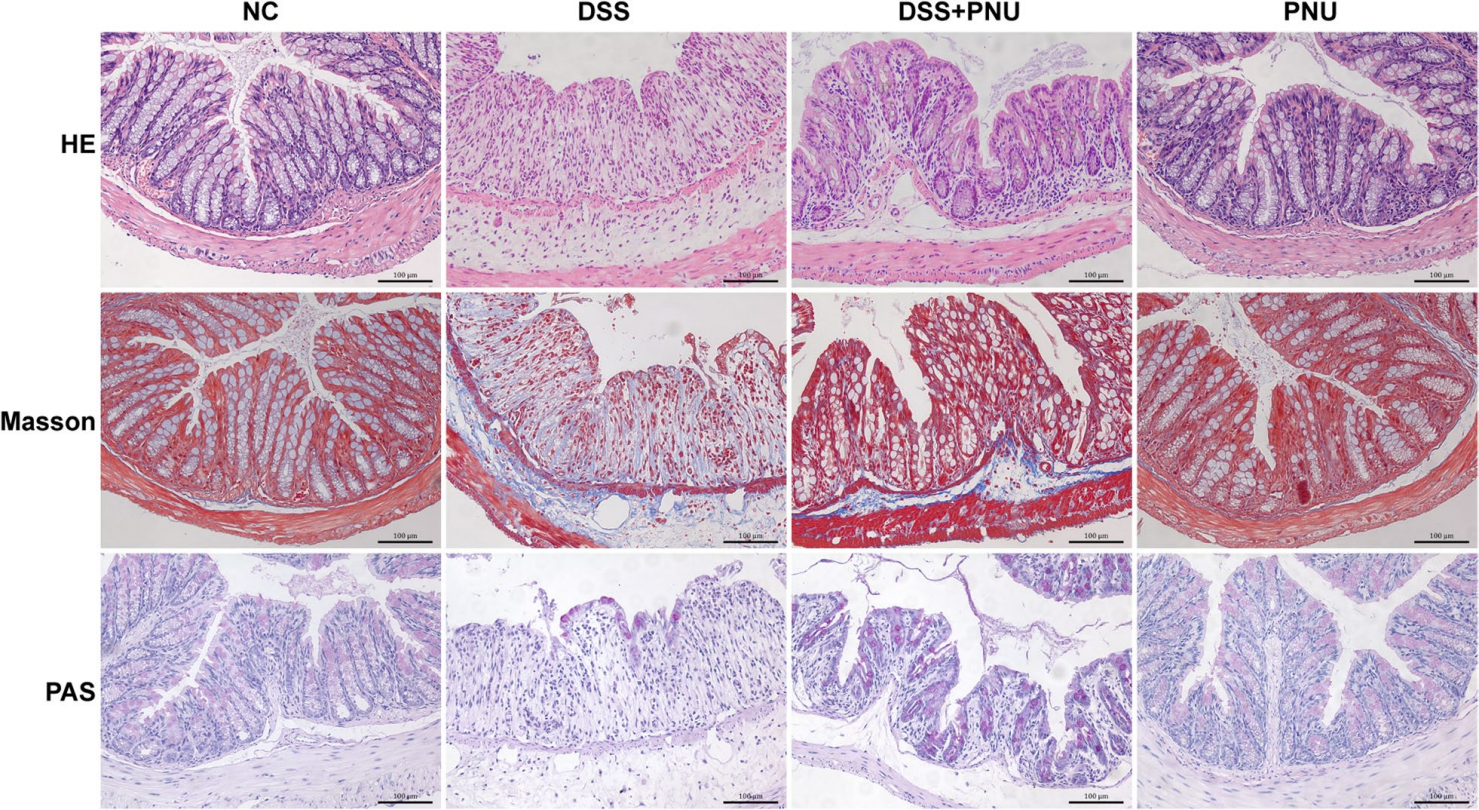
Effects of PNU treatment on colon tissue injury in DSS-induced chronic colitis mice. Effect of PNU-282987 treatment on the macroscopic and histologic manifestations of DSS colitis. Representative images of the colonic tissues from the control, colitis group (3% DSS), PNU treatment colitis group (3% DSS + PNU) and PNU treatment colitis group. Formalin-fixed, paraffin-embedded 5 μm cross-sections were stained with hematoxylin and eosin (H&E), Masson and periodic acid-Schiff staining (PAS). Scale bar: 100 μm

**Fig. 5:**
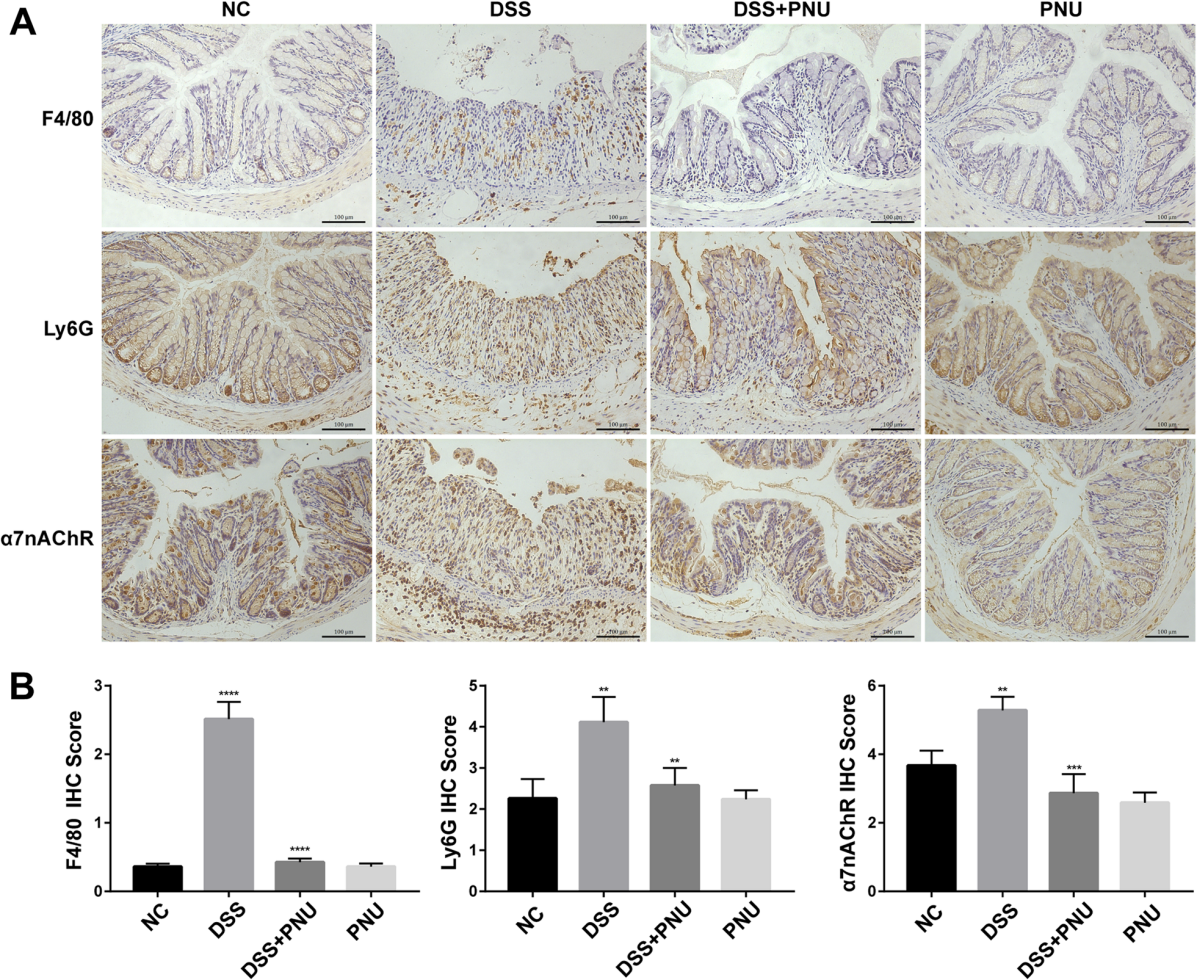
Effects of PNU on the colonic infiltration of inflammatory cells in DSS-colitis mice. **A** Representative images and IHC scores of the colonic tissues from the control, IBD (3% DSS), PNU (3% DSS + PNU) and PNU groups. The expression levels of F4/80, Ly6G and α7nAChR were examined by immunohistochemistry. Scale bar: 100 μm. **B** Immunohistochemistry protein expression score; *P < 0.05, **P < 0.01, ***P < 0.001, ****P < 0.0001

Based on the observation that PNU-282987 treatment in vivo could significantly alleviate the clinical features of experimental colitis, we next evaluated the relevant histopathological changes. H&E, Masson, and PAS staining were performed to observe the histopathological changes. As expected, compared with the healthy control group, the disease model groups developed significant histological abnormalities of the lamina propria and obvious pathological changes in the intestinal architecture (Fig. 4), and there was massive accumulation of inflammatory cells, with the presence of both mononuclear and polymorphonuclear leucocytes, increased fibrosis, disruption of intestinal mucosa, muscle layer thickening, epithelial ulcerations, crypt loss and depletion of goblet cells, and loss of intestinal architecture (Fig. 4). In vivo administration of the α7nAChR agonist PNU-282987 to mice significantly reduced colon fibrosis, accompanied by restoration of the colonic architecture and re-epithelialization. This improvement was followed by the recovery of the normal morphology of goblet cells (Fig. 4). In response to α7nAChR agonist PNU-282987 treatment, inflammatory cell infiltration was significantly decreased, and the number of F4/80-positive stained macrophages and Ly6G-positive stained neutrophils were detected by immunohistochemistry compared with the experimental groups (Fig. 5A and B). The expression pattern of α7nAChR in the colonic tissue was also examined. As shown in Fig. 5, there was an increased intensity of α7nAChR-positive cells in the experimental colitis groups compared with the normal healthy control. PNU-282987 treatment significantly decreased the expression of α7nAChR in the colonic tissue because there were increased numbers of α7nAChR-positive cells observed by immunohistochemistry staining (Fig. 5A and B).

### α7nAChR activation inhibited NF-κB and MAPK pathway activation in the colon of DSS-induced colitis mice

**Fig. 6:**
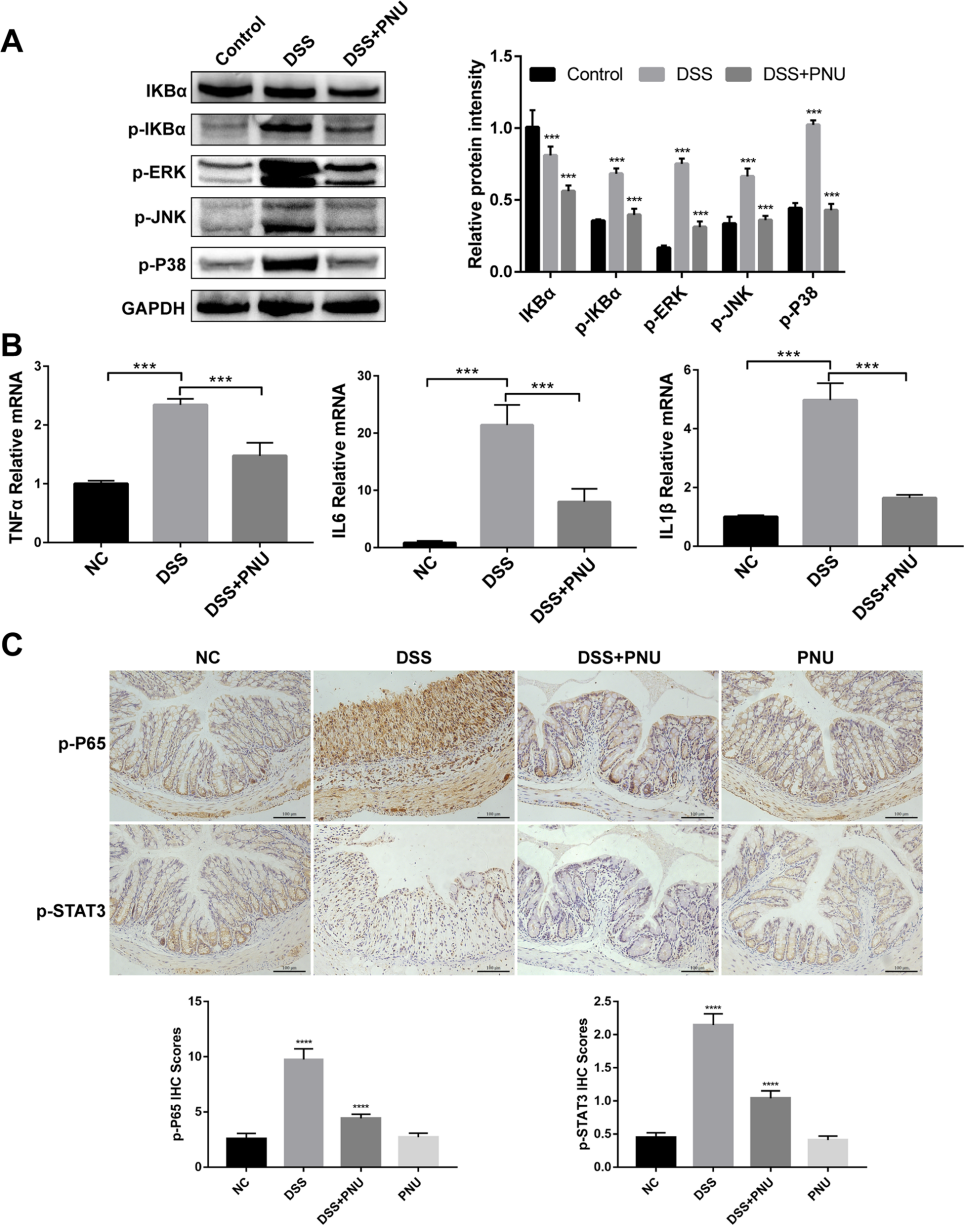
Effects of the PNU agonist on activated MAPK and NF-κB signaling pathways and mRNA expression of proinflammatory cytokines in DSS-induced colitis. Experimental colitis was induced by 3% DSS. **A** Total colon tissue protein was extracted to measure the expression of NF-κB and MAPK signaling proteins by Western blot with antibodies specific for IKBα, p-IKBα, p-ERK, p-JNK and p-P38. The housekeeping gene GAPDH was used as a protein loading control; **B** Mouse colon mRNA was extracted to determine the mRNA abundance of TNFα, IL6 and IL1β by RT–qPCR. The mRNA expression levels of the cytokines in the mice that received various treatments were normalized to the levels in control mice, which were set as 1. Data are expressed as the mean ± SD; *P < 0.05, **P < 0.01, ***P < 0.001; **C** The expression levels of NF-κB and p-STAT3 were examined by immunohistochemistry. Scale bar: 100 μm

Based on the finding that treatment with the α7nAChR agonist PNU-282987 could potentially inhibit DSS-induced colonic inflammatory responses, to address the underlying mechanism, two key signaling pathways, NF-κB and MAPK, which play roles in colitis pathogenesis and are reported to be involved in CAP bioactivity, were next assessed (Yang 2015). Through colon tissue analysis by Western blot, we found that PNU-282987 treatment significantly downregulated the level of phosphorylated forms of IκB (p-IκBα) and the activation of the NF-κB signaling pathway, and MAPK pathway activation was also inhibited (Fig. 6A). DSS treatment triggered the phosphorylation of ERK, JNK, and P38 proteins, which were significantly alleviated upon treatment with PNU-282987 compared with the DSS or control group (Fig. 6A).

Further analysis of the mRNA expression levels of these proinflammatory cytokines in the colon was performed by RT–qPCR. The expression of TNF-α, IL-6 and IL-1β was substantially upregulated in DSS-induced colitis mice, and treatment with PNU-282987 significantly reduced the enhancement (Fig. 6B). We also detected the expression of p-P65 and p-STAT3 in intestinal tissue by immunohistochemistry staining (IHC). The results showed that the increased expression of p-P65 and p-STAT3 in experimental colitis was significantly blocked upon PNU-282987 treatment (Fig. 6C). These data indicated that PNU treatment was capable of regulating intestinal inflammatory infiltrate and improving tissue architecture, which was disrupted in DSS-induced colitis.

### In vitro α7nAChR activation alleviated TLR4-mediated inflammation in macrophages

**Fig. 7:**
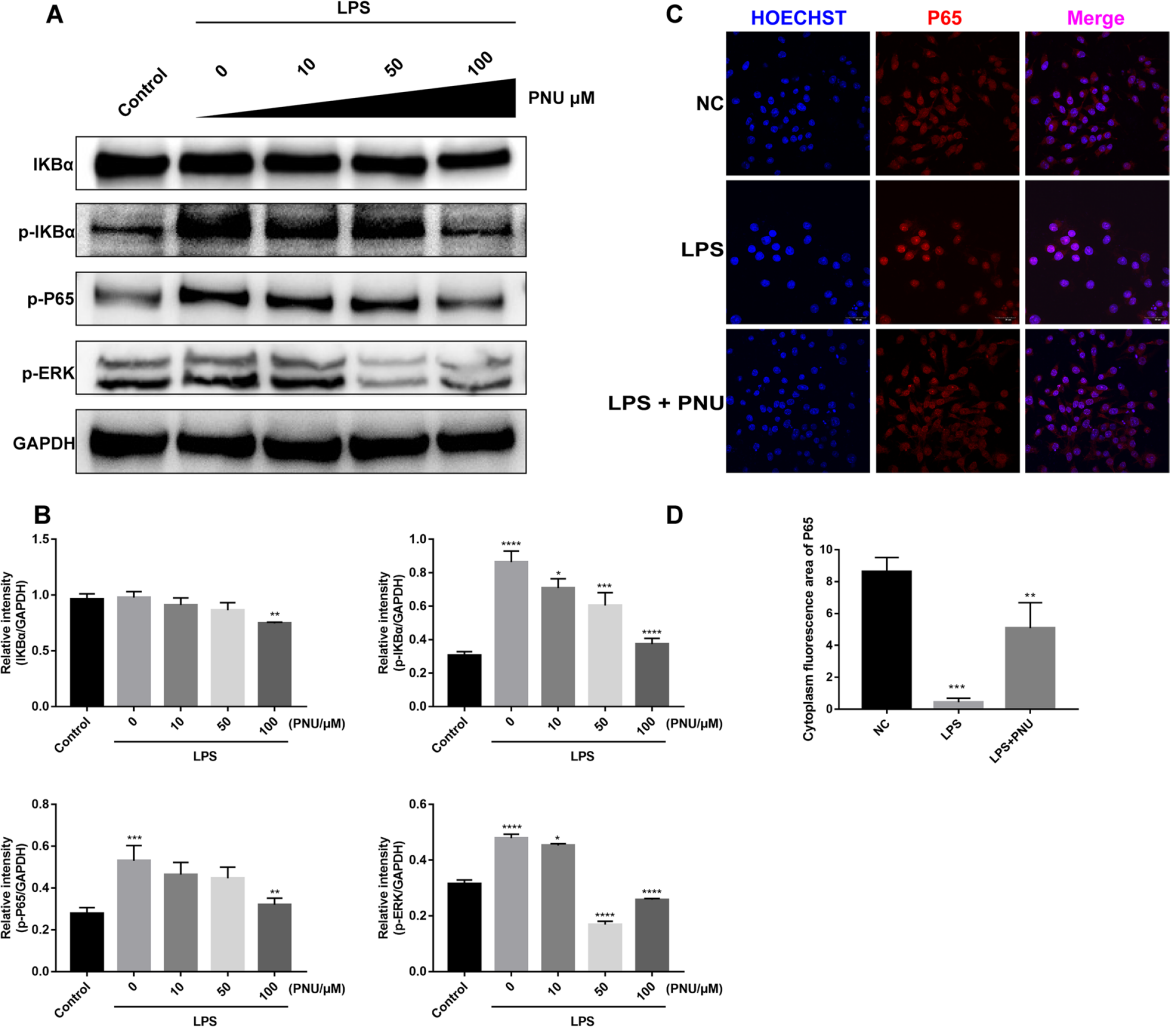
PNU decreased the expression levels of NF-κB and MAPK in RAW264.7 cells. The expression levels of MAPK and NF-κB pathway-related proteins were detected by Western blot. **A** Western blot assays were performed to detect the protein expression levels of IKBα, p-IKBα, p-P65, p-ERK and GAPDH in RAW264.7 cells. **B** Relative Western blot protein intensity. **C** The effect of PNU-282987 on the nuclear translocation of NF-κB p65 was measured by immunofluorescence. Blue fluorescence represents the cell nucleus, and red fluorescence represents p65. The p65 subunit translocated into the nucleus after LPS stimulation; PNU reduced p65 nuclear translocation caused by LPS (scale bar: 50 μm). **D** Immunofluorescence staining quantitative relative area

To delineate the mechanism of α7nAChR activation in inflammation regulation, RAW264.7 cells were preexposed to the specific α7nAChR agonist PNU-282987 before LPS challenge, and inflammatory-related pathways, including NF-κB p-p65, p-IκBα and p-ERK, were assessed. Our results showed that the protein levels of NF-κB p-p65, p-IκBα, and p-ERK were significantly downregulated in the LPS alone treatment group but were significantly decreased upon PNU-282987 pretreatment. Dose-dependent effects were observed when the PNU-282987 concentration was increased (Fig. 7A and B). Immunofluorescence staining for p65 revealed that LPS induced p65 nuclear translocation, and PNU-282987 pretreatment reversed this effect (Fig. 7C and D). Altogether, these results show that blocking NF-κB signaling pathway activation is likely an important mechanism for PNU-282987-mediated anti-inflammatory effects.

### α7nAChR activation reduced the M1/M2 ratio in the colon of DSS-induced colitis mice

**Fig. 8:**
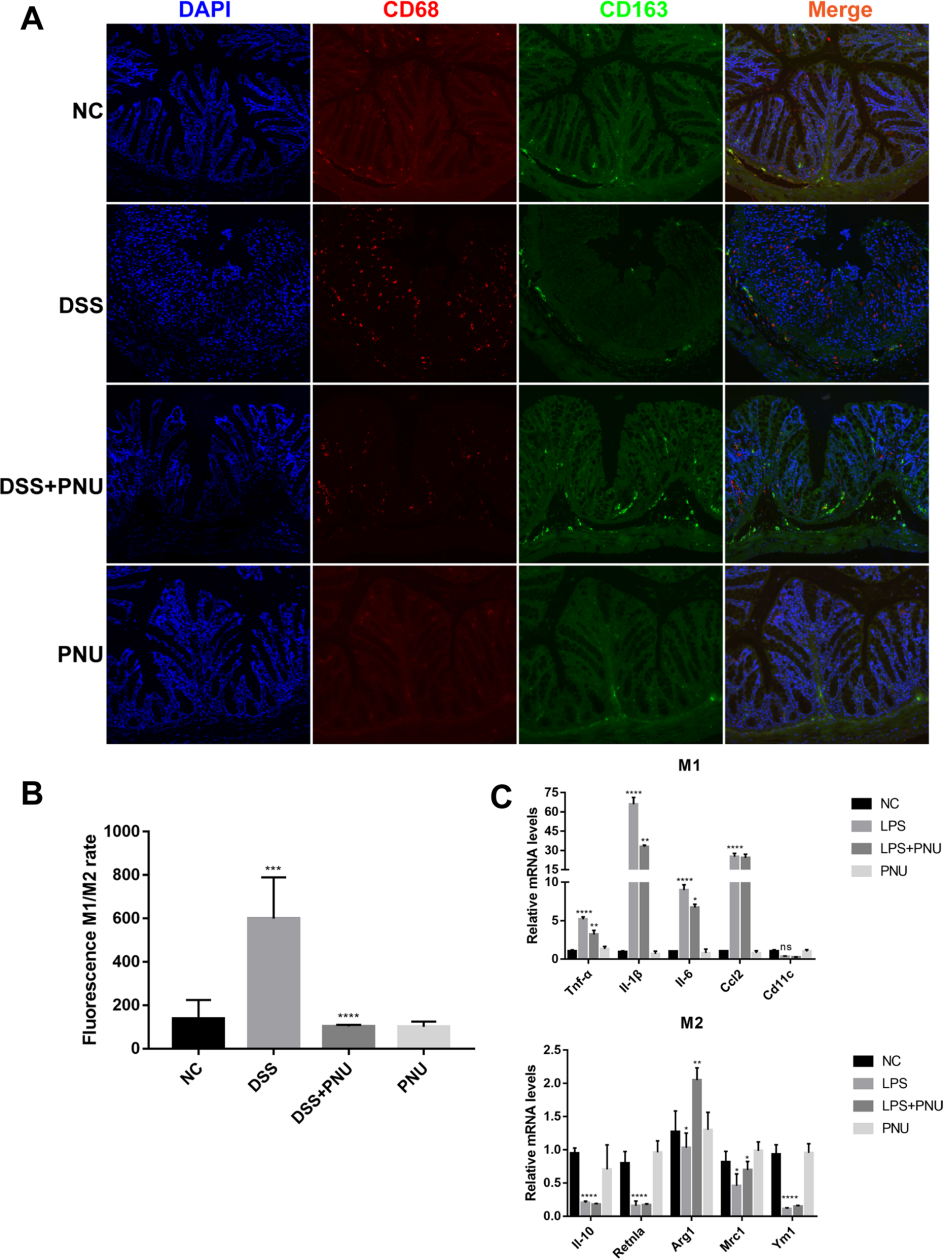
PNU decreased the ratio of M1 macrophages in colon tissues of mice and RAW264.7 cells. The M1/M2 ratio of macrophages was detected by immunofluorescence and qPCR. **A** Immunostaining assays were performed to detect the ratio of M1/M2 in colon tissues. Scale bar, 100 μm. **B** Immunofluorescence staining of the M1/M2 quantitative relative ratio. **C** RT–qPCR was used to determine the mRNA abundance of TNFα, IL6, IL1β, Ccl2, Cd11c, IL10, Retnla, Arg1, Mrc1 and Ym1 in RAW264.7 cells. The mRNA expression levels of the cytokines in the mice that received various treatments were normalized to the levels in control mice, which were set as 1. Data are expressed as the mean ± SD; *P < 0.05, **P < 0.01, ***P < 0.001, ****P < 0.0001

After studying the potential mechanism by which α7nAChR activation inhibits DSS-induced colitis, we next explored the regulatory effect of PNU-282987 on macrophage function in colitis, and the changes in the M1 and M2 phenotypes of macrophages in colitis were evaluated. By analyzing colon tissue with immunofluorescence staining, we found that PNU-282987 treatment significantly decreased the ratio of M1/M2 macrophages and that M1 macrophages decreased. In response to PNU-282987 treatment, we detected a significantly decreased number of CD68^+^M1 macrophages compared with the colitis model group, while the number of CD68^+^CD163^+^ M2 macrophages did not change, and the overall ratio of M1/M2 macrophages decreased significantly (Fig. 8A and B).

RT–qPCR assays further confirmed this result. Analyzing the transcriptional expression of M1 and M2 macrophage-related markers demonstrated that there were significant increases in the expression of M1-related cytokines and chemokines, including TNF-α, IL-6, IL-1β and Ccl2, in LPS-treated RAW264.7 macrophages, and PNU-282987 treatment significantly reversed these effects (Fig. 8C). Meanwhile, we noticed that the expression levels of Arg1- and Mrc1-related markers in M2 macrophages increased after PNU-282987 treatment (Fig. 8C). These data suggest that PNU-282987 treatment can regulate the activation type of intestinal macrophages and significantly inhibit the production of M1 proinflammatory macrophages.

### Selective agonist PNU-282987 inhibited LPS-induced phagocytosis of macrophages in RAW264.7 cells

**Fig. 9:**
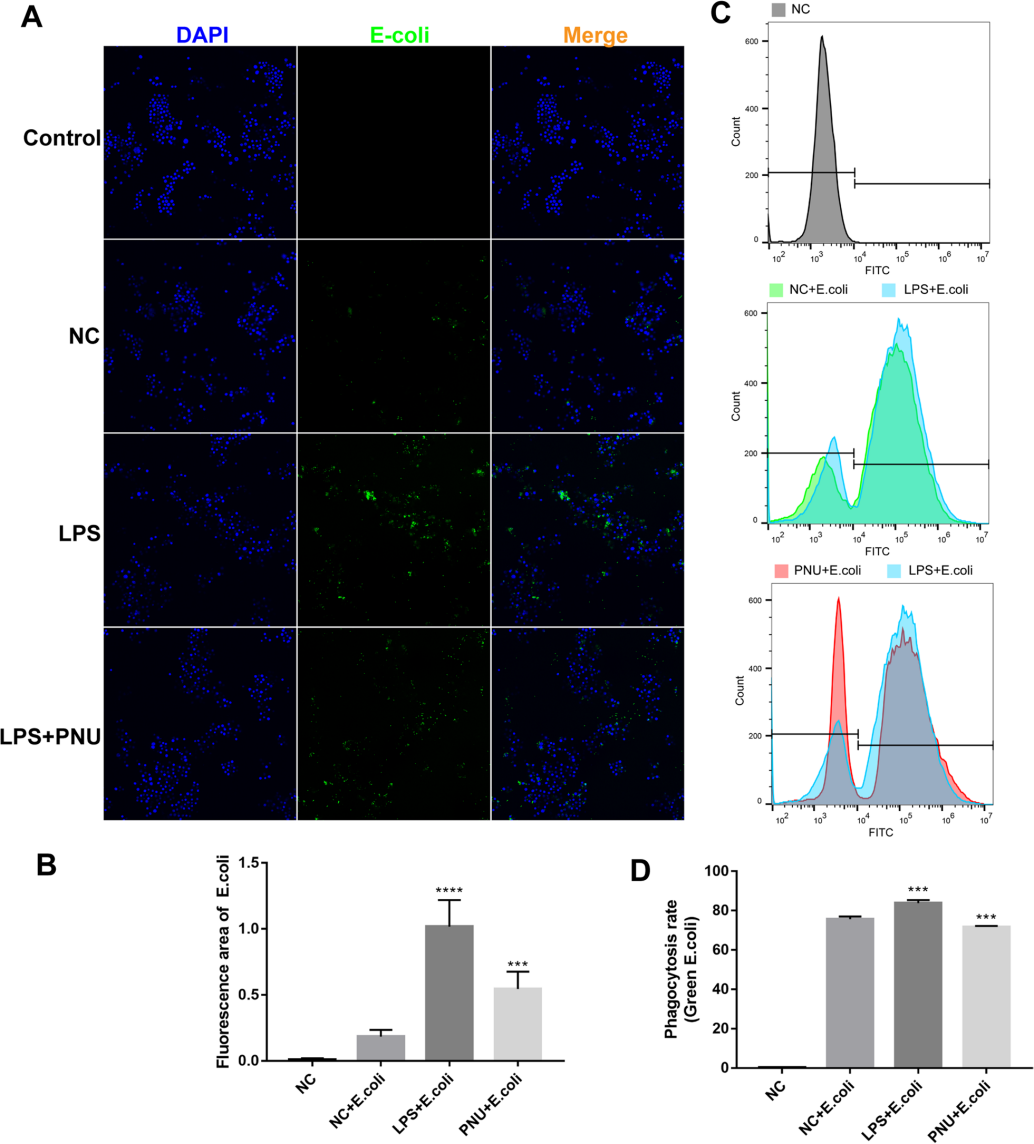
PNU decreased the phagocytosis of macrophages in colon tissues of mice and RAW264.7 cells. The phagocytic function of macrophages was detected by immunofluorescence and flow cytometry. **A** Immunostaining assay was performed to detect *E-coli* phagocytosis of macrophages in RAW264.7 cells. Scale bar, 100 μm. **B** Immunofluorescence staining quantitative relative area. **C** Phagocytosis was analyzed by flow cytometry. **D** The rate of phagocytosis was calculated using the median fluorescence intensity (MFI). Data are expressed as the mean ± SD; *P < 0.05, **P < 0.01, ***P < 0.001, ****P < 0.0001

To clarify, α7nAChR activation may affect phagocytosis by macrophages. LPS-induced RAW264.7 cells were treated with the α7nAChR agonist PNU-282987, and macrophage phagocytosis was assessed with *E-coli*. Our immunofluorescence results showed that LPS-induced macrophage phagocytosis increased significantly, but it decreased significantly following PNU-282987 treatment (Fig. 9A and B). Flow cytometry assays also confirmed these data. The activation of α7nAChR reduced the phagocytosis of *E-coli* by macrophages (Fig. 9C and D). These results suggest that treatment with PNU-282987 can inhibit the activation of macrophage phagocytosis and might inhibit the activity of proinflammatory macrophages. The specific biological significance needs to be further explored.

## Discussion

IBD is considered a recurrent and prolonged intestinal disorder resulting from chronic inflammation and tissue destruction via aberrant activation of various signaling pathways of inflammation and overexpression of multiple types of proinflammatory molecules (Park 2017). A recent study indicated that there is a direct correlation between gastrointestinal inflammation and neuronal disorders. In the current study, α7nAChR activation was found to play a crucial role in the regulation of the pathological development of IBD. Our study demonstrated that selective α7nAChR agonist PNU-282987 treatment could alleviate intestinal inflammation in a DSS-induced colitis mouse model, manifested by amelioration of acute colonic injuries and restoration of the DAI scores. Treatment with PNU-282987 in vivo was able to reduce the loss of crypts, goblet cells, and mucosal damage at the histopathological level; therefore, the therapeutic effect of α7nAChR agonist in a colitis mouse model was suggested.

The central nervous system cooperates dynamically with the immune system to regulate inflammation through humoral and neural pathways. In particular, acetylcholine (ACh), the main neurotransmitter in the vagus nerve, is dependent on α7nAChRs and could influence the outcome of the immune response via the cholinergic anti-inflammatory pathway, which represents an important homeostatic regulatory mechanism for sensing and controlling the body's response to inflammatory stimuli (Xie 2020). α7nAChRs, which are expressed not only on neural cells but also on various types of immune cells, sense acetylcholine (ACh) and mediate anti-inflammatory effects. Additionally, a growing amount of evidence shows that the brain-gut axis plays an important role in the pathogenesis of inflammation in IBD patients (Mogilevski 2019). The involvement of α7nAChRs in the pathological development of intestinal inflammation was first reported in a vagotomy mouse model, and it was found that vagotomy increased the DAI, macroscopic and histological scores, MPO activity, and proinflammatory cytokine levels in colitis colon tissue in TNBS or DSS induced acute colitis mouse models (Ghia 2006). Our study provided direct experimental evidence showing that activation of the cholinergic anti-inflammatory pathway by the selective α7nAChR agonist PNU-282987 could alleviate intestinal inflammation in a DSS-induced colitis mouse model.

Recruitment of immune cells, such as monocytes and neutrophils, to the gut epithelium contributes to the pathogenesis of experimental IBD. In our study, we demonstrated that compared with the experimental colitis model group, PNU-282987 treatment significantly reduced the number of infiltrated macrophages and neutrophils, which were identified based on F4/80 and Ly6G staining. Additionally, we observed significantly increased expression of the acetylcholine receptor (α7nAChR) in the colitis mouse model group, which indicated that the activation of the cholinergic anti-inflammatory pathway seems to be an important self-protective approach for maintaining immune homeostasis and protecting against inflammation-induced tissue damage.

Tremendous activation of multiple types of proinflammatory factors, including TNF-α, IL-1β, and IL-6, has been found to participate in the pathological development of colitis, and within this process, the activation of multiple types of signaling pathways (Tracey 2002), such as NF-κB, MAPK, COX-2, and Jak/Stat, regulates the pathological development of this disease (Liu 2005). The deficiency of the TNF-α receptor or IL-6 in mice causes less severe inflammation upon DSS exposure, suggesting that these molecules and related signaling pathways play a key role in the pathogenesis of colitis (Popivanova 2008). Stimulation of the cervical vagus has been found to inhibit the release of inflammatory factors. In an LPS-induced septic peritonitis mouse model, direct stimulation of the cervical vagus could significantly decrease the expression level of proinflammatory factors such as TNF-α in the serum, heart and liver, suggesting its anti-inflammatory and immunoregulatory functions (Westerloo 2005). In our study, we confirmed this finding, and our results showed that activation of α7nAChR with PNU-282987 significantly downregulated the expression of multiple types of proinflammatory factors, including TNF-α, IL-6, and IL-1β.

Macrophages represent the first line of host defense, which is important for maintaining intestinal immune homeostasis. Macrophages can effectively discriminate innocuous antigens and dangerous pathogens to maintain oral tolerance. Macrophage polarization from the M1 to M2 phenotype and aberrant activation of the NF-κB and MAPK) pathways have been found to contribute to the pathological development of IBD. Some studies have found that the recovery of the polarization of macrophages from an M1-type proinflammatory phenotype to an anti-inflammatory phenotype could alleviate experimental colitis (Qi 2019). Other studies have reported that some therapeutic compounds exhibit strong anti-inflammatory effects in experimental colitis, mainly relying on the regulation of NF-κB and MAPK pathway activation in macrophages. Due to the heterogeneous nature of IBD, some clinical studies also report that macrophage-specific NF-κB activation dynamics can segregate IBD patients into groups with different phenotypes and may help determine the response to therapy (Tang). Therefore, blocking the aberrant activation of the NF-κB and MAPK signaling pathways while maintaining the anti-inflammatory phenotype of macrophages has pivotal roles in the prevention and treatment of IBD. Previous reports have shown that stimulation of α7nAChR in macrophages reduced nuclear translocation of nuclear factor κB (NF-κB) through containment of the Jak2/STAT3/Socs3 signaling pathways (Jonge 2005). This provides strong evidence for the involvement of central cholinergic receptors in the anti-inflammatory pathway. In our study, we confirmed that activation of α7nAChR cholinergic receptors with PNU-282987 significantly downregulated LPS-induced activation of the NF-κB and MAPK pathways in macrophages. Consistently, our data also showed that the expression of p-IKBα, p-P65 p-ERK, p-JNK and p-P38 was notably increased in DSS-induced mouse colitis. Treatment with PNU-282987 markedly decreased the expression of p-IKBα, p-P65, p-ERK, p-JNK and p-P38 in mice with DSS-induced experimental colitis. Finally, we also verified the effect of PNU-282987 on macrophage function. The results showed that PNU-282987 treatment significantly reduced the accumulation of M1 proinflammatory macrophages and the M1/M2 ratio. Combined with in *vitro* experiments, it inhibited the decrease in macrophage phagocytosis induced by LPS, indicating that PNU-282987 may improve IBD by reducing M1 macrophages and inhibiting the function of proinflammatory macrophages.

## Conclusion

Our present study demonstrated that PNU-282987 could ameliorate pathological gut injury in a DSS-induced acute colitis mouse model, and an overall improvement in both macroscopic and histological injury was observed upon treatment with the α7nAChR agonist PNU-282987. The anti-inflammatory function of PNU-282987 may depend on the NF-κB and MAPK signaling pathways. We confirmed that the activation of the cholinergic anti-inflammatory pathway has promising potential for the treatment of IBD, which provides a pivotal approach toward a future treatment option for this disease.

## Data Availability

The corresponding author can provide the data that support the findings of this study upon request.

## Abbreviations

CAP: Cholinergic anti-inflammatory pathway
CD: Crohn’s disease
DAI: Disease activity index
DMEM: Dulbecco’s modified Eagle’s medium
DSS: Dextran sulfate sodium
H&E: Hematoxylin and eosin
IBD: Inflammatory bowel disease
MAPK: Mitogen-activated protein kinase
MFI: Median fluorescence intensity
NK: Natural Killer
PAS: Periodic acid-Schiff staining
TLR: Toll-like receptors
UC: Ulcerative colitis
IBD: Inflammatory bowel disease
Ach: Acetylcholine
α7nAChR: Nicotinic acetylcholine receptors of α7 subtype
NF-κB: Nuclear factor κB
